# Effects of Cold Stress and Ammonia Concentration on Productive Performance and Egg Quality Traits of Laying Hens

**DOI:** 10.3390/ani10122252

**Published:** 2020-11-30

**Authors:** Dapeng Li, Qin Tong, Zhengxiang Shi, Weichao Zheng, Yu Wang, Baoming Li, Geqi Yan

**Affiliations:** 1 Department of Agricultural Structure and Bioenvironmental Engineering, College of Water Resources & Civil Engineering, China Agricultural University, Beijing 100083, China; lidp@cau.edu.cn (D.L.); tongqin@cau.edu.cn (Q.T.); weichaozheng@cau.edu.cn (W.Z.); wyu@cau.edu.cn (Y.W.); libm@cau.edu.cn (B.L.); yangeqi@cau.edu.cn (G.Y.); 2 Key Laboratory of Agricultural Engineering in Structure and Environment, Ministry of Agriculture and Rural Affairs, Beijing 100083, China; 3 Beijing Engineering Research Center on Animal Healthy Environment, Beijing 100083, China

**Keywords:** cold stress, ammonia, productive performance, egg quality, laying hen

## Abstract

**Simple Summary:**

In the cold season, in order to alleviate the adverse effect of the low temperature, restricting or reducing ventilation is one of the common methods to preserve indoor heat to ensure the suitable temperature for hens in many poultry houses with no heating equipment. However, ventilation is the most important means to discharge ammonia in the house and ensure the indoor air quality in poultry farms. Measure of limiting ventilation to save energy usually causes the accumulation of gaseous ammonia released by poultry manure, which results in an increased indoor ammonia concentration. Therefore, studying the performance of laying hens exposed to a long-term ammonia concentration is of great significance for ventilation management in winter. In addition, it is not clear how the interaction between low temperature and ammonia concentration affects the performance of laying hens. Therefore, this study aims to evaluate the effects of temperature, ammonia and their interaction on productive performance and egg quality of laying hens breeding from the start to the peak period of laying. The study is of great significance to the poultry industry regarding the ventilation management in winter from the perspective of controlling temperature and ammonia concentration. It is also a reference for ensuring layer productive performance and egg quality.

**Abstract:**

In a cold climate, ensuring indoor air quality and heat preservation simultaneously has always been a difficult problem in the poultry house. The current study was carried out in order to determine the effects of chronic low temperature and ammonia concentration on productive performance and egg quality of commercial laying hens. 576 18-week-old Hy-line Brown hens were used in this study. Birds were housed in cages and received for 20-week exposure to low temperature and ammonia in six artificial environmental chambers. Birds were randomly assigned into six treatments: treatment 1 (T1, 20 °C, ≤5 ppm, control group), treatment 2 (T2, 20 °C, 20 ppm), treatment 3 (T3, 20 °C, 45 ppm), treatment 4 (T4, 8 °C, ≤5 ppm), treatment 5 (T5, 8 °C, 20 ppm) and treatment 6 (T6, 8 °C, 45 ppm). Daily feed intake (DFI), feed efficiency (FE), egg production (EP) and body weight (BW) were recorded and calculated from 19 weeks of age. Egg samples were collected at 22, 26, 30, 34 and 38 weeks of age and egg weight (EW), shell breaking strength (SBS), albumen height (AH), yolk weight (YW), shell weight (SW), shell thickness (ST) and Haugh unit (HU) were measured. The results of the present study indicated that low temperature and excessive ammonia decreased the EP of hens compared with those of the T1 birds. Low temperature increased DFI of hens thereby FE showed significant differences among treatments. During the early period of the experiment, low temperature treatment increased the BW of laying hens, but this trend of increase was suppressed by the treatment of ammonia with the prolongation of the experimental period. Egg quality was also affected by low temperature and excessive ammonia. At different experimental periods, egg quality traits of hens exposed to the cold and ammonia stress presented significant differences compared to those of control birds. The present study indicated that the effect of ammonia was more pronounced on hens than that of low temperature at the early and peak laying period in terms of several main traits of productive performance and egg quality under long term hens breeding.

## 1. Introduction

Poultry are exposed to various environmental stressors including adverse climatic conditions, harmful gases, dust and infectious diseases throughout their lives [1,2]. It has been demonstrated that the health and welfare of animals are markedly affected by low ambient temperature [3,4]. The thermal neutral zone for the metabolic and productive activity is around 18–23.9 °C for adult laying hens [5]. Similarly, it was reported that the optimum temperature for the thermoneutral zone was between 19 and 22 °C for laying hens [6]. However, the ambient temperature varies from –5 to +5 °C in many regions of the world during winter months [7,8]. Cold environmental temperatures below 16°C result in significant adverse effects on animal’s productive performance. For birds, such cold environment conditions can lead to a variety of negative effects such as increased feed intake, decreased egg production and nutrient digestibility, affect individual body weight and feed efficiency and so on [7,9,10]. Egg quality is one of the most crucial factors influencing the bird’s reproductive function and economic profitability of the poultry industry. It has been reported that low ambient temperature reduced egg quality traits by decreasing egg weight, eggshell quality, Haugh unit, albumen height and yolk index [5,8,11,12,13].

As is well known, it is not energy-saving and economical to heat animal buildings. The measures to mitigate the adverse effects of cold stress have mainly focused on enhancing energy intake and feed additives including minerals and vitamin supplements such as vitamin C and chromium [2,7,8,11,12,13,14]. Additionally, restricting ventilation is also one of the common-used methods to retain indoor heat. However, this attempt to conserve energy in winter usually lead to the accumulation of ammonia released by poultry manure, which may result in a high level of atmospheric ammonia [15]. Gaseous ammonia is considered to be one of the most toxic gases in poultry houses, and its adverse effects on the environment have been proven by many previous studies [16,17,18]. Meanwhile, the poultry industry has to concern not only the worker’s health, but also the welfare of bird. Since the elevated concentration of atmospheric ammonia has a negative influence on bird physiology and productive performance [19,20,21,22,23]. The exposure to ammonia leads to the markedly influences on health and growth performance of poultry. Excessive ammonia was recognized as a kind of environment stressors for birds that reduced feed intake and stunt their growth [24,25,26,27,28]. Furthermore, it decreased egg production significantly for 7 weeks of exposure to ammonia at the concentration of 102 ppm [24] and adversely affected egg quality [29]. On the contrary, average body weights of broilers were not significantly affected by ammonia concentration of 0, 25 or 50 ppm for the 49-d treatment [30]. Similarly, Deaton et al. [15] reported that no significant differences were found in egg weight, feed efficiency and egg shell strength of 12-week-old layers between the control group and the ammonia exposure groups (100 ppm) after the 17-d treatment. Controversial results indicated that ammonia exposure lowered productive performance and the egg quality of birds, but the results may depend on bird age and ammonia level and duration.

Compared with heat stress, there are fewer studies on the effect of cold stress on laying hens, while studies on the effect of ammonia on poultry are mostly focused on broilers. As it is well known, laying hens are kept longer in the hen house than broilers [25]. With the modernization of environmental management, the control of ammonia concentration in the layer house has been greatly improved compared with the past. Ammonia concentration as high as 100 ppm is almost impossible to appear. Therefore, studying the productive performance and egg quality of laying hens exposed to relatively low ammonia concentrations for an extended period is of great significance to layer houses. It was proved that birds naturally adapted more easily to lower temperature than to higher temperature [31]. Whether birds show adaptability or not under the long-term effects of these two factors is unknown. Moreover, it is not clear that if these two factors have an interaction on productive performance and egg quality of laying hens. Therefore, this work was conducted to evaluate the effect of low temperature, ammonia concentration and the interaction between them on productive performance and egg quality traits of laying hens from the beginning to the peak laying period. If possible, suggestions can be made for the winter indoor ventilation management from the perspective of saving energy and maintaining productivity based on this investigation.

## 2. Materials and Methods

### 2.1. Experimental Rooms for Bird Rearing

**Figure 1:**
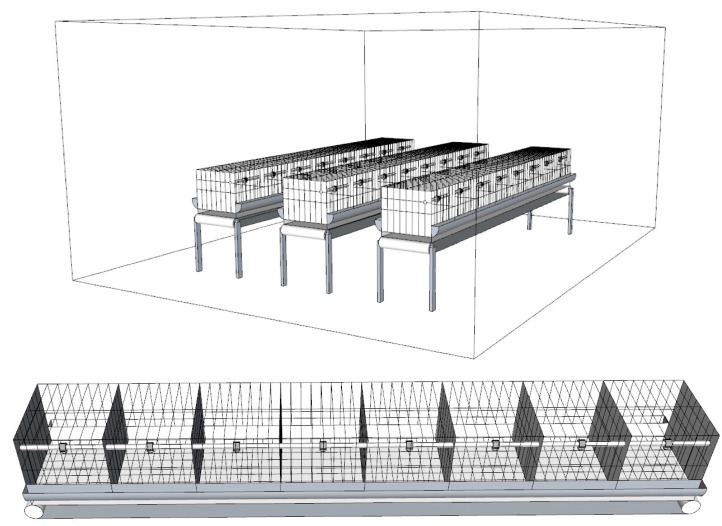
Arrangement of cages in each experimental room.

Six identical artificial environmental rooms in terms of size (each 24.8 m^2^, 4.5 m × 5.5 m, L × W), equipment and sensors were used for the experiment. Bird cages (50 cm × 40 cm × 40 cm, L × W × H) were divided into 3 groups (8 cages/group) and were distributed evenly to each room for birds breeding (Figure 1). Each experimental room was programmed by a computer to control environmental factors (temperature, relative humidity, gas concentration and light intensity) as required. Environment sensors were installed in each room to monitor the data of temperature, relative humidity, concentration of NH_3_ and CO_2_ every 10 s and real-time displayed on the controller screen for observation. LED lamps on the ceiling of the room could be manually adjusted to provide required light intensity. The program system analyzed the temperature information collected by the sensors and controlled the air conditioner to keep the indoor temperature. Air inlet and outlet were trepanned and fans were fitted for automatic mechanical ventilation. Feeder and drinker were provided in each room.

### 2.2. Experimental Design and Animal Management

All the hens used in the current study were cared following the protocols by the Laboratory Animal Ethical Committee of China Agricultural University. This animal study was approved by Laboratory Animal Ethical Committee of China Agricultural University on 1st June 2018 (ethic code: 20180036). A total of 576 laying hens (Hy-Line Brown) obtained from a commercial poultry farm were used for the current study. All laying hens were randomly divided into six groups and placed in cages (4 birds/cage). Corn-soybean meal-based commercial layer diets were provided. Hens were provided with feed and water ad libitum. Birds took 1-week environment acclimation in each experimental room at 18 weeks of age before temperature and NH_3_ exposure. As shown in Table 1, the experimental setup based on a total of five treatments with 2 temperatures (20 ± 2 °C, 8 ± 2 °C) and 3 ammonia concentrations (≤5 ppm, 20 ± 2 ppm, 45 ± 2 ppm) and a control group (20 ± 2 °C, ≤5 ppm). T1 was the control group. There were 3 replications (32 birds/replicate) in each room.

Ammonia tester was purchased from Yaoan Electronic Technology Development Co., Ltd. Shandong Province, China. Its ammonia sensor (model CLE-1012-401) was provided by Honeywell. According to the instrument, its test range is 0–100 ppm, sensitivity is 0.135 ± 0.0035 μA/ppm, accuracy is 0.5 ppm, working temperature range is −20–40 °C and working humidity is 15–90% RH. The ammonia tester was assembled and programmed by the above company. As for the ammonia concentration test, every corner (total 8) of each rooms and 3 points for each row of cages on the height of the cage were tested for 24 h before this experiment. The air relative humidity (RH) was maintained at 40–60% and the CO_2_ level was limited below 1000 ppm during the experiment. The lighting regime during the experimental period from 18 weeks of age was 12 h/day (4:00–16:00) and then it was prolonged gradually every week until 16 h (4:00–20:00) at 31 weeks of age. From then on, it turned to permanent illumination of 16 h until the end of this experiment. Besides, light intensity of 30 lx was set at the bird head level. For other details of the bird management and experiment design, please refer to the article published by the authors [32].

### 2.3. Sample Collection

Feed consumption of hens and total number of eggs laid by hens were recorded weekly in each replication. Meanwhile, for each treatment, 24 hens (8 hens/replication) were selected randomly and weighed for individual body weight (BW) after fasting for 12 h starting at 22, 26, 30, 34 and 38 weeks of age. Besides, 24 eggs (8 eggs/replication) were collected randomly for the egg quality test at 22, 26, 30, 34 and 38 weeks of age. All egg samples were stored at 4 °C.

### 2.4. Measured Contents and Methods

For productive performance, daily feed intake (DFI) of hens was determined by dividing the weekly feed consumption by the number of chickens and then dividing this data by 7. The egg production (EP) was calculated by separating the number of weekly picked up eggs by the number of hens on the same week. Feed efficiency (FE) was calculated by dividing the feed consumption by the egg mass produced during the time that feed consumption was measured. As to egg quality determination, eggs were weighed and recorded for egg weight (EW). Shell breaking strength (SBS) was evaluated by an eggshell force gauge (EFR-01, ORKA FOOD TECHNOLOGY LTD., Bountiful, UT, USA). Then eggs were cracked onto a level surface and the albumen height (AH) was measured by an egg quality analyzer (EA-01, ORKA FOOD TECHNOLOGY LTD., Bountiful, UT, USA) after which yolk was isolated and weighed for yolk weight (YW). The egg shells were washed, dried and then weighed for shell weight (SW). Next, shell thickness (ST) was detected by calculating the thickness mean values measured at three locations on the egg (air cell, equator of egg point and sharp end) using a thickness tester (ESTG-01, ORKA FOOD TECHNOLOGY LTD., Bountiful, UT, USA) after the inner membrane has been removed. Haugh unit (HU) value was calculated by the formula as followed: HU = 100 × log(7.57 − 1.7EW^0.37^ + AH). All operations referred to the product manual provided by the company.

### 2.5. Statistical Analysis

Linear mixed models parameterized with SPSS (IBM SPSS Statistics 25.0, Armonk, NY, USA) were used for statistical analyses. Firstly, all the data were analyzed for the homogeneity of the variances. Main effects of temperature, ammonia concentration, week of age and fixed effects of 2-way interactions between them and the random effect of replicate were evaluated. The model is shown in the following formula:

Y_ijku_ = μ + T_i_ + AC_j_ + WOA_k_ + R_u_ + T × AC_ij_ + T × WOA_ik_ +AC × WOA_jk_ + ε_ijku_,

where Y_ijku_ = traits (DFI, FE, EP, BW, EW, YW, SW, ST, SBS, AH and HU) investigated; μ = model constant; T_i_ (temperature) = effect of temperature (i = 1 to 2); AC_j_ (ammonia concentration) = effect of ammonia concentration (j = 1 to 3); WOA_k_ (weeks of age) = effect of age (k = 1 to 5); R_u_ (replicate) = effect of replicate (u = 1 to 3); T × AC_ij_ = effect of interaction between T and AC; T × WOA_ik_ = effect of interaction between T and WOA; AC × WOA_jk_ = effect of interaction between AC and WOA and ε_ijku_ = the residual error term.

Effects of the factor were removed from the original model when they were not significant. The differences were considered significant at *p* ≤ 0.05. Data were analyzed by 1-way ANOVA and post-hoc group comparisons were performed by Duncan’s multiple range (least significant difference) test. Results were presented as mean ± SE.

## 3. Results

The statistical analysis results showed that for all parameters investigated, the effect of the replicate was not significant. Therefore, it was excluded from the above model.

### 3.1. Productive Performance

As shown in Table 2, temperature, ammonia concentration and bird age had significant effects on DFI, FE, EP and BW of laying hens (*p* < 0.05). A significant 2-way interaction between ammonia concentration and age was found for DFI, FE, EP and BW while the 2-way interaction between temperature and ammonia concentration and temperature and age for BW was not significant. DFI, FE and BW of hens were significantly increased with temperature decreasing. On the contrary, the EP of layers was significantly decreased under 8 °C treatment (*p* < 0.05). Compared with the treatment of low ammonia concentration (≤5 ppm), all the productive performance parameters were significantly reduced under the ammonia concentrations of 20 ppm and 45 ppm (*p* < 0.05). Furthermore, the BW of hens increased steadily with the age (*p* < 0.05). However, the other parameters increased first and then decreased with the age.

**Figure 2:**
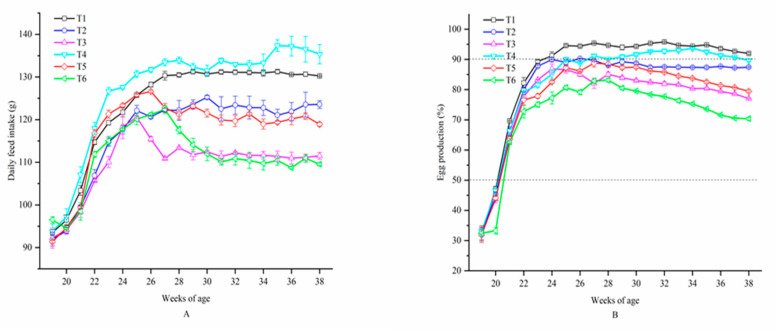
Daily feed intake and egg production of laying hens exposed to graded levels of ammonia under 20 °C and 8 °C at 22, 26, 30, 34 and 38 weeks of age. **A** = daily feed intake; **B** = egg production. T1 = treatment 1 (20 °C, ≤ 5 ppm, control group), T2 = treatment 2 (20 °C, 20 ppm), T3 = treatment 3 (20 °C, 45 ppm), T4 = treatment 4 (8 °C, ≤ 5 ppm), T5 = treatment 5 (8 °C, 20 ppm) and T6 = treatment 6 (8 °C, 45 ppm). Data represent mean ± SE.

Figure 2 presents that the DFI of birds in T6 was significantly more than that of birds in T1–T5 at the age of 19 weeks (*p* < 0.05). As the experiment went on, hens in T4 took more food than hens in other treatments. Little change was observed in DFI of birds in T1, T2 and T5 from 27 to 38 weeks of age, while DFI of birds in T6 and T3 decreased and significant difference was found among the six treatments at 38 weeks of age. A significant lower EP was observed in T6 compared to the other treatments at the age of 20 weeks (*p* < 0.05). At 21 weeks of age, the EP of laying hens in all treatments exceeded 50% and the EP of hens in T1 was over 90% at 24 weeks of age. However, the EP of birds in T2, T3, T5 and T6 never reached 90% and they decreased from 29 weeks of age on. Hens in T1 produced more eggs than those in other treatments from 20 weeks of age on.

**Figure 3:**
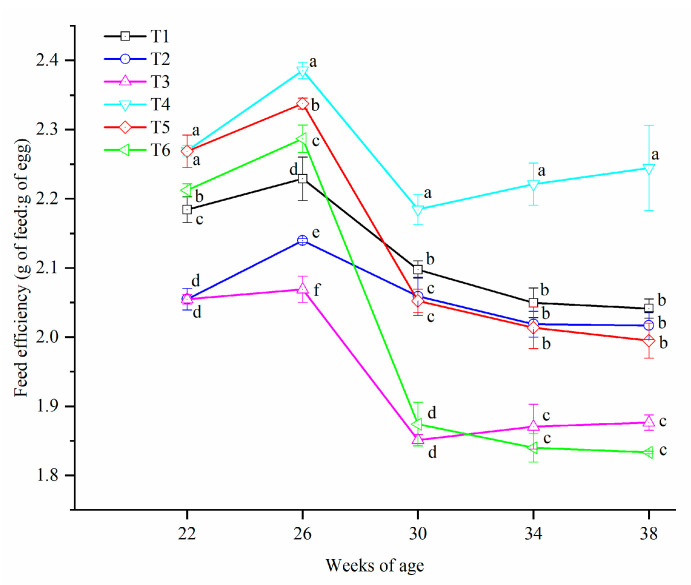
Feed efficiency of laying hens exposed to graded levels of ammonia under 20 °C and 8 °C at 22, 26, 30, 34 and 38 weeks of age. T1 = treatment 1 (20 °C, ≤ 5 ppm, control group), T2 = treatment 2 (20 °C, 20 ppm), T3 = treatment 3 (20 °C, 45 ppm), T4 = treatment 4 (8 °C, ≤ 5 ppm), T5 = treatment 5 (8 °C, 20 ppm) and T6 = treatment 6 (8 °C, 45 ppm). Data represent mean ± SE. ^a–f^ Values marked with different letters are significantly different (*p* < 0.05).

The FE of birds in all treatments increased to the maximum at the age of 26 weeks and then decreased until the end of this experiment (Figure 3). Significant differences were found at every sampling week (*p* < 0.05) and hens in T4 showed the highest FE from 26 weeks of age onwards.

**Figure 4:**
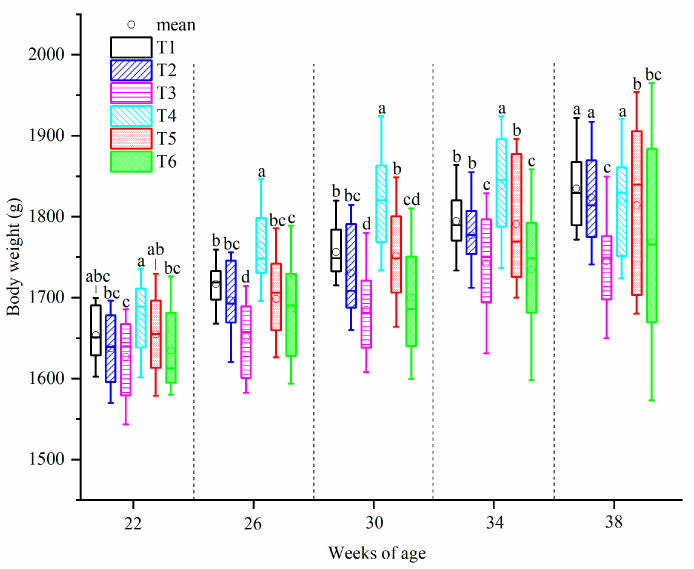
Body weight of laying hens exposed to graded levels of ammonia under 20 °C and 8 °C at 22, 26, 30, 34 and 38 weeks of age. T1 = treatment 1 (20 °C, ≤ 5 ppm, control group), T2 = treatment 2 (20 °C, 20 ppm), T3 = treatment 3 (20 °C, 45 ppm), T4 = treatment 4 (8 °C, ≤ 5 ppm), T5 = treatment 5 (8 °C, 20 ppm) and T6 = treatment 6 (8 °C, 45 ppm). Data represent mean ± SE. ^a–d^ Values marked with different letters are significantly different (*p* < 0.05).

Changes in BW of laying hens are presented in Figure 4. The BW of hens in all treatments kept growing except those in T3 and T4 during the experimental period. Significant differences among the treatments were found at every sampling week (*p* < 0.05) while no significant difference was observed between T1 and T2.

### 3.2. Egg Quality Traits

Table 3 presents that all egg quality traits were significantly affected by temperature, ammonia concentration and weeks of age (*p* < 0.05) and 2-way interactions between them on egg quality traits were found (*p* < 0.05) except the interactions between T × AC and AC × WOA on SBS. All egg quality traits were significantly decreased with decreased temperature (*p* < 0.05). Furthermore, all the traits were significantly decreased with the increased ammonia concentrations (*p* < 0.05). However, no significant difference in SW was found between birds exposed to 20 ppm and those in 45 ppm treatment. EW, YW and ST increased with the age and significant differences were found among different temperatures, ammonia concentrations and weeks of age (*p* < 0.05). On the contrary, SBS decreased with age. Values of SW, AH and HU peaked at 26, 30 and 26 weeks of age (*p* < 0.05) compared to that of 22 weeks of age and decreased until 38 weeks of age.

**Figure 5:**
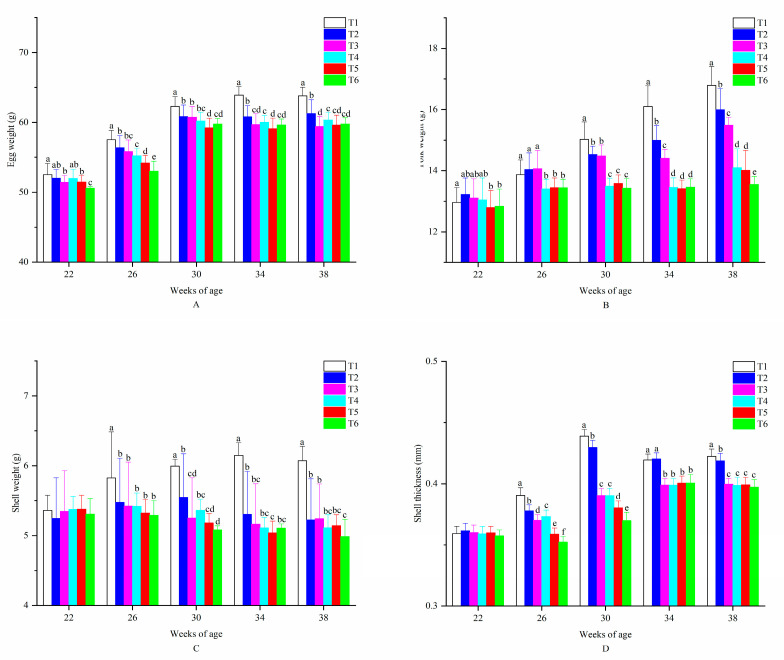
Egg quality traits of laying hens exposed to graded levels of ammonia under 20 °C and 8 °C at 22, 26, 30, 34 and 38 weeks of age. (**A**) egg weight; (**B**) yolk weight; (**C**) shell weight and (**D**) shell thickness. T1 = treatment 1 (20 °C, ≤ 5 ppm, control group), T2 = treatment 2 (20 °C, 20 ppm), T3 = treatment 3 (20 °C, 45 ppm), T4 = treatment 4 (8 °C, ≤ 5 ppm), T5 = treatment 5 (8 °C, 20 ppm) and T6 = treatment 6 (8 °C, 45 ppm). Data represent mean ± SE. ^a–f^ Values marked with different letters are significantly different (*p* < 0.05).

As presented in Figure 5, significant differences in EW between the birds in the control group (T1) and those exposed to ammonia treatment under the low temperature (T5 and T6) were found at all sampling weeks (*p* < 0.05). Furthermore, no significant difference was found between low ammonia concentration treatment and moderate ammonia concentration treatment by comparing T1 and T2, T4 and T5 at the age of 22 weeks. However, the above situation changed at 26 weeks of age. Hens in the control group led a heaviest EW during the whole experimental period. No significant differences were found between the control and ammonia treatments (T2 and T3) until 30 weeks of age (*p* < 0.05). From the age of 30 weeks, YW of eggs produced by hens in T1 was markedly heavier (*p* < 0.05) than those in the other treatments (T2–T6) until the end of this experiment. No significant difference was observed in SW between the six treatments at the age of 22 weeks. As the experiment went on, hens in the control group showed a significant heavier SW (*p* < 0.05) than those in the other treatments (T2–T6). The SW of hens in T1-T4 trended to increase with age and then decreased, different from those in T2, which decreased from the first sampling week. Similarly to SW, no significant difference was found in ST at 22 weeks of age. ST values of birds in T1 and T2 were significantly larger than those in other treatments from 26 weeks of age (*p* < 0.05). In addition, no significant differences in ST were found among T3, T4, T5 and T6 since 34 weeks of age.

**Figure 6:**
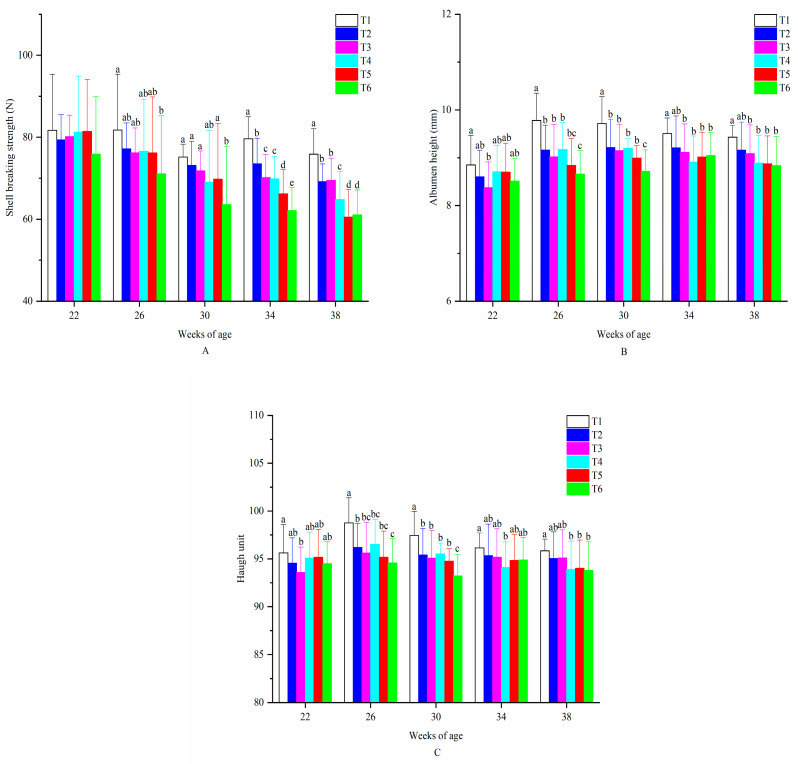
Egg quality traits of laying hens exposed to graded levels of ammonia under 20 °C and 8 °C at 22, 26, 30, 34 and 38 weeks of age. (**A**) shell breaking strength; (**B**) albumen height; (**C**) Haugh unit. T1 = treatment 1 (20 °C, ≤ 5 ppm, control group), T2 = treatment 2 (20 °C, 20 ppm), T3 = treatment 3 (20 °C, 45 ppm), T4 = treatment 4 (8 °C, ≤ 5 ppm), T5 = treatment 5 (8 °C, 20 ppm) and T6 = treatment 6 (8 °C, 45 ppm). Data represent mean ± SE. ^a–e^ Values marked with different letters are significantly different (*p* < 0.05).

Birds in all six treatments showed no significant difference in SBS at the age of 22 weeks (Figure 6). All the hens tended to have a decreased SBS with age. Additionally, at the age of 26 and 30 weeks, birds in T6 had a significantly lower value of SBS than those in T1–T5 (*p* < 0.05). Hens in the control group led a largest SBS during the sampling weeks. AH of birds in T1 was significantly higher than in the other treatments at the age of 26 and 30 weeks (*p* < 0.05) while this difference was not significant at the next two sampling weeks. Furthermore, no significant differences were observed between T1 and T2, which was similar to the situation among T3, T4, T5 and T6. Birds in the control group had the highest AH values all the time. The changes of HU value and differences between the treatments were very closed to those of AH.

## 4. Discussion

In the present study, the effects of low ambient temperature and different ammonia concentrations on productive performance in terms of daily feed intake, egg production, feed efficiency and body weight and egg quality in terms of egg weight, yolk weight, shell weight, shell thickness, shell breaking strength, albumen height and Haugh unit of laying hens reared for 20 weeks were investigated. The results indicated that the productive performance and egg quality of laying hens were affected by low ambient temperature, ammonia concentration and age, and there were interactions between them.

Results of this study showed that the DFI of laying hens under low ambient temperature (T4–T6) increased by 1.76 g compared to those in 20 °C treatments during the 20-week experiment. Sahin and Sahin [14] presented that 32-week-old laying hens reduced their feed consumption by comparing to the birds in low ambient temperature of 6.2 °C, which was close to the result of the present research. On the other hand, DFI of hens in medium and high ammonia concentration treatments (20 and 45 ppm) were reduced by 8.28 g and 16.36 g respectively compared to that of laying hens in clean air treatments in the present study. Charles and Payne [24] found that continuous exposure of birds to ammonia (105 ppm) reduced feed intake by 10.4%. The reducing effect of ammonia on DFI in our experiment was larger than that of the study operated by Deaton et al. [33], which treated laying hens for a 17-day ammonia exposure. This difference may be due to the longer duration of the current experiment. Furthermore, the decrease of DFI caused by elevated ammonia was greater than that of low temperature according to the results of the present study. Hens under low temperature had 16.67–25.84 g higher DFI than those in high ammonia concentration treatments from 26 to 38 weeks of age, which indicated that excessive ammonia may decrease the DFI while cold stress may increase the DFI [16,24]. Meanwhile, the results of DFI in ammonia treatments under low temperature indicated that the combination of these two factors might aggravate the reduction of bird’s DFI.

Different effects of various environmental factors on the feed conversion ratio of birds had been reported. It was reported that excessive ammonia affected the level of reproductive hormones in the blood, which may cause a decrease in the production performance of laying hens [32]. Ammonia at a concentration of up to 50 ppm during the first 4 weeks of age may reduce feed efficiency and over 7–8 weeks resulted in a decreased appetite of birds, which in turn resulted in a reduced efficiency of food utilization [30,34]. On the contrast, treating birds with low temperature of 6 °C can increase the feed efficiency of birds [10,11]. In other words, FE of birds was affected by multiple factors. In the present study, FE of hens in all six treatments increased with age until 26 weeks of age and then tended to decrease, which may be explained as follows: The increase in FE at the beginning of the experiment may be due to the influence of low temperature, while the decrease in FE at the late stage of the experiment may be mainly caused by the excessive concentration of ammonia. As the experiment progressed, the influence of ammonia concentration on FE was getting bigger than low temperature. In the present study, FE of birds in high ammonia concentrations under low temperature was 10.3% lower than that of birds in the control group at the age of 38 weeks. This result may be because the interaction between low temperature and ammonia reduced feed consumption (FC), egg production and egg weight mass (EWM), causing a decreased FC/EWM ratio. Although birds in two treatments (T3 and T6) had a lower FE value, it did not mean that the laying hens of these two treatments were in good health condition or had a better productive performance, because FE was determined by two factors: feed intake and egg weight [35,36]. In the present study, the EP of laying hens treated with 8 °C was 4.08% lower than that of laying hens in normal temperature, which agreed with the studies of Sahin et al. [8] on laying hens treated with 6.8 ± 3 °C for 15 weeks and Akbari et al. [13] on laying hens exposed to the same temperature for 8 weeks. Similarly, laying hens in 20 ppm and 45 ppm treatments produced 5.29% and 11.99% fewer eggs than laying hens in clean air treatments (≤5 ppm), respectively. It was reported that the EP of hens exposed to a 17-d ammonia concentration treatment of 200 ppm was reduced by 6.3% and hens treated with 28-d 100 ppm ammonia level produced 2.9% fewer eggs than those in fresh air [15]. Under the combined effects of low temperature and elevated ammonia concentration, the reduction of laying hens’ egg production in the low temperature and high ammonia concentration (45 ppm) treatments reached a maximum of 22.06% at 37 weeks of age compared to the control group while birds in the low temperature treatment (T4) presented a close EP to the control group, which indicated that both medium (20 ppm) and high (45 ppm) levels of ammonia gas exacerbated the decrease of egg production during the 20-week exposure and birds showed adaptability to low temperature in terms of egg production. Ensminger et al. [37] demonstrated that birds are naturally well adapted to cold mainly due to their highly efficient insulation provided by feathers. Exposures to low temperature and excessive ammonia caused a prolonged time to reach 50% and 90% hen’s egg production, which agrees with the statement of David et al. [38].

The present study showed that low temperature increased the BW of birds for 18.82 g after the 20-week experiment compared to that of those in normal temperature while low and high ammonia exposure decreased the BW of birds (30.15 g and 70.22 g, respectively) compared to that of those in fresh air. The study of Quarles et al. [39] reported that the 8-week body weights of broilers exposed to 25 and 50 ppm ammonia were significantly lighter than those of broilers not subjected to ammonia. However, the average body weight of birds (1881 g, 1862 g and 1805 g respectively) treated with 0, 25 and 50 ppm concentrations of ammonia for 49 days did not differ significantly [30]. When the surrounding temperature fell below 18 °C, the body may not be able to warm itself and would take more food to produce heat and fat [4]. These findings were consistent with the results of the current study. Moreover, the greater errors of BW mean values of birds exposed to low temperature and a high ammonia level may indicate that different individuals responded differently to environmental stressors in terms of body weight.

In this study, egg weight was decreased by medium (20 ppm) and high (45 ppm) concentrations of ammonia for 1.29 g and 1.79 g respectively. This is in contrast to the result of a study on laying hens exposed to 100 ppm and 200 ppm for 17 days and 28 days, respectively [15], which was not surprising because the experimental period of the current study was much longer than this previous study. Cold stress also reduced the egg weight of birds according to the current and previous research [2,5,11]. Shell quality traits (SW, ST and SBS) were adversely influenced by low ambient temperature and ammonia, respectively. Deaton et al. [15] also reported that hens subjected to 100 ppm and 200 ppm levels of ammonia had a worse egg shell quality. This may be explained by the effect of temperature on the blood calcium level of birds, because calcium is a main element in eggshell shaping [40]. Only minor changes were seen in the shell quality traits with age at the late period of this experiment in all environments because the hens have difficulty producing an increased amount of egg shell at an older age [41]. Eggs laid by hens in low temperature had a lower albumen height, yolk weight and Haugh unit indicating that the low temperature had an adverse effect on the egg quality. However, it was reported that the exposure of 100 ppm could be tolerated for short periods without an immediate drastic loss in laying performance and egg quality [15], which differed from our study. The adverse effects of ammonia on egg quality may be related to the exposure time, bird strain and age and ammonia concentration.

## 5. Conclusions

The present study indicated that the effect of ammonia was more pronounced on hens than that of low temperature at the early and peak laying period in terms of several main traits of productive performance and egg quality under long term hens breeding. It suggests that limiting ventilation to ensure the suitable temperature should better not be considered as the only/paramount measure during the winter time when no heating equipment is available for the poultry house to achieve heat preservation. Appropriate ventilation to reduce the ammonia concentration was more conducive to the performance of laying hens. According to the results of the present study, the negative impact of ammonia on laying hens is more serious than low temperature, especially during long-term breeding. Further studies on low temperature and ammonia interactions on productive performance and egg quality of hens at the late reproductive period are needed.

## Figures and Tables

**Table 1:** Experimental treatments.

Treatments ^1^						
Conditions ^2^	T1	T2	T3	T4	T5	T6
T (°C)	20	20	20	8	8	8
AC (ppm)	≤5	20	45	≤5	20	45

^1^ T1–T6 = treatment 1-treatment 6; ^2^ T = temperature; AC = ammonia concentration.

**Table 2:** Productive performance traits of laying hens exposed to graded levels of ammonia under 20 °C and 8 °C at 22, 26, 30, 34 and 38 weeks of age.

Productive Performance Traits ^1^				
Conditions ^2^	DFI (g)	FE	EP (%)	BW (g)
T (°C)				
20	119.37 ± 8.58 ^b^	2.04 ± 0.11 ^b^	86.32 ± 5.94 ^a^	1724.62 ± 78.45 ^b^
8	121.13 ± 8.61 ^a^	2.13 ± 0.18 ^a^	82.24 ± 6.97 ^b^	1743.44 ± 93.25 ^a^
AC (ppm)				
≤5	128.46 ± 6.54 ^a^	2.19 ± 0.10 ^a^	90.04 ± 5.16 ^a^	1767.49 ± 78.02 ^a^
20	120.18 ± 5.44 ^b^	2.10 ± 0.11 ^b^	84.75 ± 4.46 ^b^	1737.34 ± 84.66 ^b^
45	112.10 ± 3.99 ^c^	1.98 ± 0.16 ^c^	78.05 ± 4.36 ^c^	1697.27 ± 82.63 ^c^
WOA				
22	112.30 ± 4.89 ^c^	2.17 ± 0.09 ^b^	78.11 ± 3.28 ^d^	1647.16 ± 46.29 ^e^
26	123.97 ± 5.58 ^a^	2.24 ± 0.11 ^a^	87.32 ± 4.88 ^a^	1702.20 ± 57.51 ^d^
30	122.23 ± 8.09 ^b^	2.02 ± 0.12 ^c^	87.46 ± 5.14 ^a^	1740.49 ± 70.99 ^c^
34	121.22 ± 9.20 ^b^	2.00 ± 0.13 ^c^	85.85 ± 7.07 ^b^	1779.47 ± 68.31 ^b^
38	121.51 ± 9.65 ^b^	2.00 ± 0.14 ^c^	82.65 ± 7.88 ^c^	1800.83 ± 85.67 ^a^
*p*-value				
T	<0.05	<0.05	<0.05	<0.05
AC	<0.05	<0.05	<0.05	<0.05
WOA	<0.05	<0.05	<0.05	<0.05
T × AC	<0.05	<0.05	<0.05	NS
T × WOA	<0.05	<0.05	<0.05	NS
AC × WOA	<0.05	<0.05	<0.05	<0.05

^a–d^ Means within the main effect without a common superscript are different at *p* < 0.05. Data represent mean ± SE. ^1^ DFI = daily feed intake; FE = feed efficiency; EP = egg production; BW = body weight; ^2^ T = temperature; AC = ammonia concentration; WOA = weeks of age.

**Table 3:** Egg quality traits of laying hens exposed to graded levels of ammonia under 20 °C and 8 °C at 22, 26, 30, 34 and 38 weeks of age.

Egg Quality Traits ^1^							
Conditions ^2^	EW (g)	YW (g)	SW (g)	ST (mm)	SBS (N)	AH (mm)	HU
T (°C)							
20	58.56 ± 4.25 ^a^	14.60 ± 1.21 ^a^	5.51 ± 0.61 ^a^	0.397 ± 0.027 ^a^	75.61 ± 8.18 ^a^	9.16 ± 0.65 ^a^	95.66 ± 2.89 ^a^
8	56.93 ± 3.76 ^b^	13.43 ± 0.54 ^b^	5.21 ± 0.22 ^b^	0.379 ± 0.020 ^b^	69.95 ± 12.87 ^b^	8.87 ± 0.53 ^b^	94.67 ± 2.61 ^b^
AC (ppm)							
≤5	58.77 ± 4.31 ^a^	14.22 ± 1.36 ^a^	5.58 ± 0.46 ^a^	0.395 ± 0.026 ^a^	75.56 ± 11.45 ^a^	9.22 ± 0.61 ^a^	95.89 ± 2.73 ^a^
20	57.48 ± 3.83 ^b^	14.00 ± 1.02 ^b^	5.29 ± 0.46 ^b^	0.391 ± 0.026 ^b^	72.65 ± 10.58 ^b^	8.98 ± 0.58 ^b^	95.04 ± 2.72 ^b^
45	56.98 ± 3.92 ^c^	13.82 ± 0.85 ^c^	5.22 ± 0.44 ^b^	0.379 ± 0.020 ^c^	70.14 ± 10.75 ^c^	8.85 ± 0.60 ^c^	94.55 ± 2.78 ^c^
WOA							
22	51.66 ± 1.26 ^c^	12.99 ± 0.59 ^e^	5.33 ± 0.37 ^b,c^	0.359 ± 0.007 ^d^	79.95 ± 11.49 ^a^	8.63 ± 0.57 ^b^	94.75 ± 2.74 ^b^
26	55.35 ± 2.03 ^b^	13.71 ± 0.52 ^d^	5.46 ± 0.50 ^a^	0.370 ± 0.014 ^c^	76.49 ± 11.85 ^b^	9.10 ± 0.66 ^a^	96.13 ± 2.99 ^a^
30	60.51 ± 1.66 ^a^	14.09 ± 0.71 ^c^	5.40 ± 0.47 ^a,b^	0.400 ± 0.026 ^b^	70.41 ± 10.62 ^c^	9.16 ± 0.54 ^a^	95.24 ± 2.54 ^b^
34	60.52 ± 2.06 ^a^	14.30 ± 1.08 ^b^	5.31 ± 0.53 ^b,c^	0.406 ± 0.012 ^a^	70.25 ± 7.87 ^c^	9.13 ± 0.56 ^a^	95.07 ± 2.69 ^b^
38	60.69 ± 2.04 ^a^	14.99 ± 12.30 ^a^	5.30 ± 0.50 ^c^	0.406 ± 0.013 ^a^	66.81 ± 7.96 ^d^	9.05 ± 0.58 ^a^	94.61 ± 2.78 ^b^
*p*-value							
T	<0.05	<0.05	<0.05	<0.05	<0.05	<0.05	<0.05
AC	<0.05	<0.05	<0.05	<0.05	<0.05	<0.05	<0.05
WOA	<0.05	<0.05	<0.05	<0.05	<0.05	<0.05	<0.05
T × AC	<0.05	<0.05	<0.05	<0.05	NS	<0.05	<0.05
T × WOA	<0.05	<0.05	<0.05	<0.05	<0.05	<0.05	<0.05
AC × WOA	<0.05	<0.05	<0.05	<0.05	NS	<0.05	<0.05

^a–e^ Means within the main effect without a common superscript are different a t *p* < 0.05. Data represent mean ± SE. ^1^ EW = egg weight; YW = yolk weight; SW = shell weight; ST = shell thickness; SBS = shell breaking strength; AH = albumen height; HU = Haugh unit, ^2^ T = temperature; AC = ammonia concentration; WOA = weeks of age.
